# First description of the female and morphological variation of *Achalinushunanensis* Ma, Shi, Xiang, Shu & Jiang, 2023 (Serpentes, Xenodermidae), with range extension of this species and *A.yunkaiensis* Wang, Li & Wang, 2019

**DOI:** 10.3897/BDJ.13.e138423

**Published:** 2025-02-17

**Authors:** Yuhao Xu, Mengci Chen, Shun Ma, Shengchao Shi, Tianyou Zhang, Shiyang Weng, Lifang Peng

**Affiliations:** 1 State Key Laboratory of Plateau Ecology and Agriculture, Qinghai University, Xining, China State Key Laboratory of Plateau Ecology and Agriculture, Qinghai University Xining China; 2 School of Ecological and Environmental Engineering, Qinghai University, Xining, China School of Ecological and Environmental Engineering, Qinghai University Xining China; 3 Chengdu Institute of Biology, Chinese Academy of Sciences, Chengdu, China Chengdu Institute of Biology, Chinese Academy of Sciences Chengdu China; 4 University of Chinese Academy of Science, Beijing, China University of Chinese Academy of Science Beijing China; 5 Hubei Engineering Research Center for Protection and Utilization of Special Biological Resources in the Hanjiang River Basin, School of Life Science, Jianghan University, Wuhan, China Hubei Engineering Research Center for Protection and Utilization of Special Biological Resources in the Hanjiang River Basin, School of Life Science, Jianghan University Wuhan China; 6 School of Plant Protection, Anhui Agricultural University, Hefei, China School of Plant Protection, Anhui Agricultural University Hefei China; 7 Tibet Plateau Institute of Biology, lhasa, China Tibet Plateau Institute of Biology lhasa China

**Keywords:** *Achalinus*, China, morphology, phylogeny, Guizhou

## Abstract

**Background:**

The odd-scaled snake genus *Achalinus*, which is widely distributed in northern Vietnam, China and Japan, is a poorly-known group of snakes. Numerous species within this genus have been described based on few specimens or only a single type specimen. *Achalinushunanensis* Ma, Shi, Xiang, Shu & Jiang, 2023 was described based on only two male specimens from Huaihua City and Changsha City, Hunan Province, China. To date, this species has only been recorded in Hunan Province and the information on females is not available. During our herpetological surveys in 2024, two *Achalinus* specimens were collected from Guizhou Province, China. We compared morphology and mitochondrial DNA sequence data of these two specimens with all the species of the genus *Achalinus*. Both datasets strongly supported classification of the adult female specimen from Dushan County to *A.hunanensis* and the adult male specimen from Xifeng County to *A.yunkaiensis*.

**New information:**

In this study, we describe the first female specimen of *A.hunanensis* in detail and provide revised diagnoses of this species based on newly-collected and examined specimens and update the distribution of *A.hunanensis* and *A.yunkaiensis*.

## Introduction

The genus *Achalinus* Peters, 1869 is the most speciose genus in the snake family Xenodermidae Gray, 1849, with 28 recognised species distributed from northern Vietnam, China to Japan (Ma et al. 2023c, Yang et al. 2023, Uetz et al. 2024). Most species of *Achalinus* are adapted to a semi-fossorial life, typically having small body sizes and inconspicuous colouration, which makes them difficult to detect in the wild. In recent years, with the advancement of field surveys and widespread DNA-barcoding efforts, the underestimated biodiversity of *Achalinus* has been gradually revealed. Since 2019, more than 20 new species of the genus *Achalinus* have been described (Wang et al. 2019, Ziegler et al. 2019, Li et al. 2020, Luu et al. 2020, Miller et al. 2020, Hou et al. 2021, Huang et al. 2021, Li et al. 2021, Ha et al. 2022, Yang et al. 2022, Ma et al. 2023b, Ma et al. 2023c, Zhang et al. 2023, Li et al. 2024, Xu et al. 2024b).

The Hunan odd-scaled Snake, *Achalinushunanensis* Ma, Shi, Xiang, Shu & Jiang, 2023, was described, based on two male specimens and is currently known only from Huaihua City and Changsha City, Hunan Province, China (Ma et al. 2023b). It can be distinguished from its congeners mainly by a combination of the following morphological characters: (1) all dorsal scales strongly keeled, 23 rows throughout the body; (2) tail relatively short, TaL/TL ratio 0.221–0.225 in males; (3) maxillary teeth 23; (4) length of suture between internasals significantly longer than that between prefrontals; (5) one loreal, subrectangular; (6) six supralabials, the 4^th^ and 5^th^ in contact with the eye; (7) the two anterior temporals in contact with the eye; (8) ventrals 163–165, subcaudals 69–72 in males, not paired (Ma et al. 2023b).

**Figure 1.**
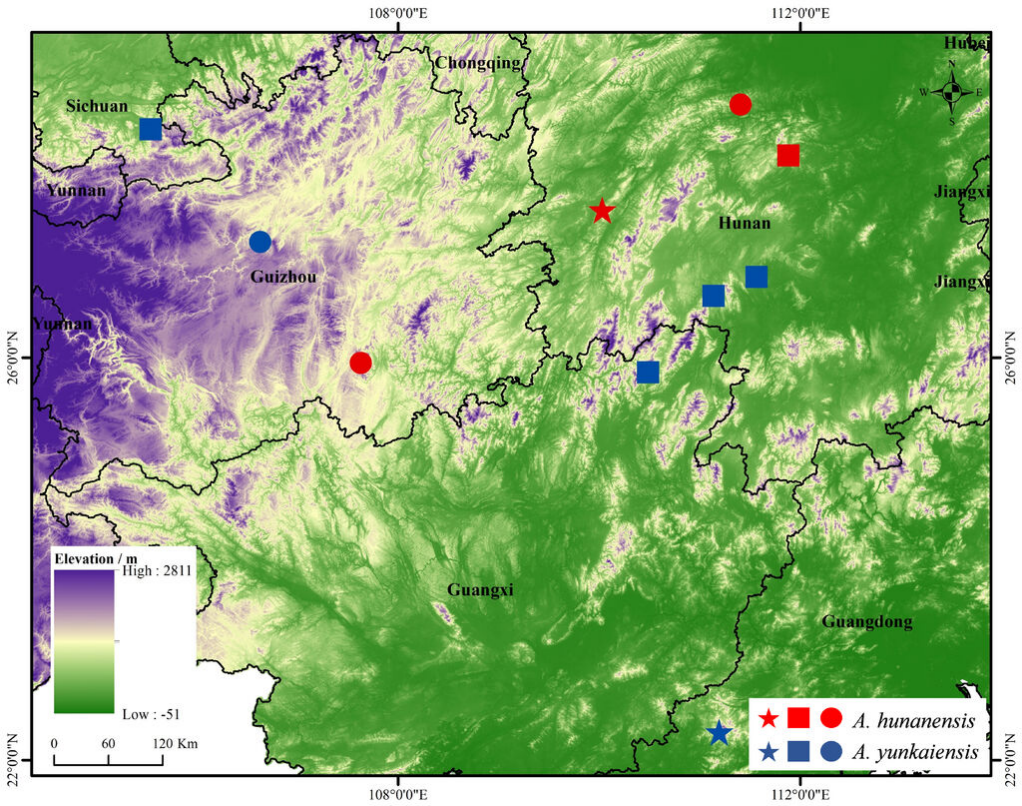
Distribution of *Achalinushunanensis* (red) and *A.yunkaiensis* (blue). The stars shows the type locality; the circles show the sampling point in this study; and the squares show the other known localities.

During our recent herpetological surveys, two *Achalinus* specimens were collected from Guizhou Province, China (Fig. 1). Molecular data revealed that the adult female specimen from Dushan County clustered with the type series of *A.hunanensis*, while the adult male specimen from Xifeng County clustered with *A.yunkaiensis*. Subsequent morphological examination supported these results. In addition, when we examined the *Achalinus* specimens deposited in the collections of Chengdu Institute of Biology (CIB), Chinese Academy of Sciences, we found an adult male specimen from Anhua County, Hunan Province which was originally identified as *Achalinusater*, but which we re-identified as *A.hunanensis*. Herein, we provide the first detailed description of the female of *A.hunanensis* and report the two new records of *A.hunanensis* and *A.yunkaiensis* from Guizhou Province.

## Materials and methods

### Sampling

Two specimens of the genus *Achalinus* were collected in Guizhou Province, China: QHU 2024027 (field number LFR2024065), an adult male, was collected in Guiyang City and QHU 2024030 (field number LFR2024120), an adult female, was collected in Qiannan Buyi and Miao Autonomous Prefecture. The specimens were humanely euthanised using a lethal injection of 0.7% tricaine methanesulphonate (MS222) solution. Fresh liver tissue was extracted and immediately preserved in 95% ethanol. The specimens were fixed in 10% formaldehyde for one day, then transferred to 75% ethanol for permanent preservation and deposited in Qinghai University Museum (QHU). All sampling procedures involving live snakes were in accordance with the Wild Animal Protection Law of China and approved by the Institutional Ethics Committee of Qinghai University (protocol code SL 2023028). Additionally, we examined a specimen deposited in the collections of the Chengdu Institute of Biology (CIB), Chinese Academy of Sciences: CIB 20160503, an adult male, was collected in Yiyang City, Hunan Province, China. The sex of all specimens was determined by gonadal dissection.

### Molecular phylogeny

Genomic DNA was extracted from preserved liver tissues using QIAamp DNA Mini Kit (QIAGEN, Changsheng Biotechnology Co. Ltd). A fragment of the mitochondrial cytochrome c oxidase subunit 1 (*CO1*) was amplified by polymerase chain reaction (PCR) using primer pairs Chmf4 and Chmr4 (Che et al. 2012). The double-stranded PCR products were sequenced by Shanghai Map Biotech Co. Ltd.

*CO1* sequences (621 bp) were assembled using SeqMan in the DNASTAR software package (Burland 2000) and compared using MEGA X software (Kumar et al. 2018). The Maximum Likelihood (ML) analysis was used with IQ-TREE 1.6.12 (Nguyen et al. 2015) to construct the phylogenetic tree. Ultrafast Bootstrap Approximation (UFB) branch support was assessed by using 5000 ultrafast bootstrap replicates and SH-like approximate likelihood ratio test (SH-aLRT) was conducted to the single branch tests by 1000 replicates. In addition, we calculated the uncorrected pairwise distances (p-distances) using the MEGA X software (Kumar et al. 2018).

For phylogenetic analysis, 32 sequences of 24 recognised species of the genus *Achalinus* were selected. Three outgroups: *Fimbriosklossi* Smith, 1921, *Parafimbrioslao* Teynié, David, Lottier, Le, Vidal & Nguyen, 2015 and *Stoliczkiavanhnuailianai* Lalronunga, Lalhmangaiha, Zosangliana, Lalhmingliani, Gower, Das & Deepak, 2021 were selected following Xu et al. (2024). All sequences were obtained from National Center for Biotechnology Information (NCBI), except the newly-generated sequences (Table 1).

### Morphological examination

Measurements and scale counts followed Zhao (2006) and Xu et al. (2024). The utilisation of abbreviations for morphological characteristics follows the conventions established by Darko et al. (2022). Three measurement characters were measured with Deli Stainless Ruler (No. 8462) to the nearest 1 mm: **SVL** (snout–vent length), **TAL** (tail length) and **TL** (total length). All other measurements were performed using Deli digital calipers (DL312200) to the nearest 0.1 mm: **HL** (head length): taken from the tip of snout to the posterior margin of mandible; **HW** (head width): measured at the widest part of the head in dorsal view; **LorH** (loreal height): measured from the highest part to the lowest part of the loreal in lateral view; **LorL** (loreal length): measured from the anteriormost to the postermost point of the loreal in lateral view; **LSBI** (length of the suture between internasals); **LSBP** (length of the suture between prefrontals); **ED** (eye diameter): taken from the anterior to the posterior edge of the eye. The scale characters and their abbreviations are as follows: **SL** (supralabials); **IL** (infralabials); **IL-1st Chin** (infralabials touching the first pair of chin shields); **Lor** (loreals); **PRO** (preoculars); **PO** (postoculars); **TEMP** (temporals); **aTEMP-Eye** (the number of anterior temporals touching the eye); **SPO** (supraoculars); **DSR** (dorsal scale rows): counted at one-head-length behind the head, at mid-body, at one-head-length before the cloacal plate; **VS** (ventrals), **CP** (cloacal plate) and **SC** (subcaudals).

## Taxon treatments

### Achalinus hunanensis

Ma, Shi, Xiang, Shu & Jiang, 2023

CC8EAD38-B53E-5677-AD24-EE4FC1A5E6D6

#### Materials

- **Type status:** Other material. **Occurrence:** catalogNumber: QHU 2024030; individualCount: 1; sex: female; disposition: in collection; associatedSequences: GenBank: PQ281493; occurrenceID: BED8699C-8929-5A3B-A5CC-B9956D6BBAB2; **Taxon:** scientificName: *Achalinushunanensis*; order: Squamata; family: Xenodermidae; genus: Achalinus; **Location:** country: China; stateProvince: Guizhou; county: Dushan; verbatimElevation: 1300 m; verbatimCoordinates: 25°56'59"N, 107°37'56"E; **Event:** eventDate: 17-08-2024
- **Type status:** Other material. **Occurrence:** catalogNumber: CIB 20160503; individualCount: 1; sex: male; disposition: in collection; associatedSequences: GenBank: PQ281494; occurrenceID: 6F555952-2708-5D5E-AD57-61B575D6017E; **Taxon:** scientificName: *Achalinushunanensis*; order: Squamata; family: Xenodermidae; genus: Achalinus; **Location:** country: China; stateProvince: Hunan; county: Anhua; verbatimElevation: 550 m; verbatimCoordinates: 28°31'02"N, 111°24'28"E; **Event:** eventDate: 03-05-2016

#### Description of the female specimen (QHU 2024030)

**Measurements and scalation.** Measurements of the female *A.hunanensis* specimen are listed in Table 2. Body slender and cylindrical; head slightly wider than neck; eye small, ED 1.2 mm; rostrum small, triangular, slightly visible from above; length of the suture between the internasal substantially longer than that between prefrontal, LSBI 1.9 mm, LSBP 0.9 mm, LSBI/LSBP ratio 2.1; nasal divided into two sections by nasal cleft, nostril in the anterior part of the nasal; prefrontals paired; frontal pentagonal, pointed to the rear, slightly wider than high, much shorter than the parietals; loreal one, subrectangular, LorH 1.0 mm, LorL 1.6 mm, LorH/LorL ratio 0.63; supraocular one, pentagonal; TEMP 7/7, arranged in three rows (2+2+3 on both sides), the anterior two contact the eye; six supralabials, the 4^th^–5^th^ contact the eye, the last one much elongated; two pairs of chin shields, the anterior pairs almost equal to the posterior pairs, followed by preventrals; one mental; 6/6 infralabials, the first one contacting with others after the mental and before the 1^st^ chin-shields, 1^st^–3^rd^ touching the first pair of chin-shields.

Dorsal scales strongly keeled, lanceolate, 23 rows throughout the body, the outermost row strongly keeled and substantially enlarged. VS 169; anal entire; SC 53, not paired.

**Figure 2.**
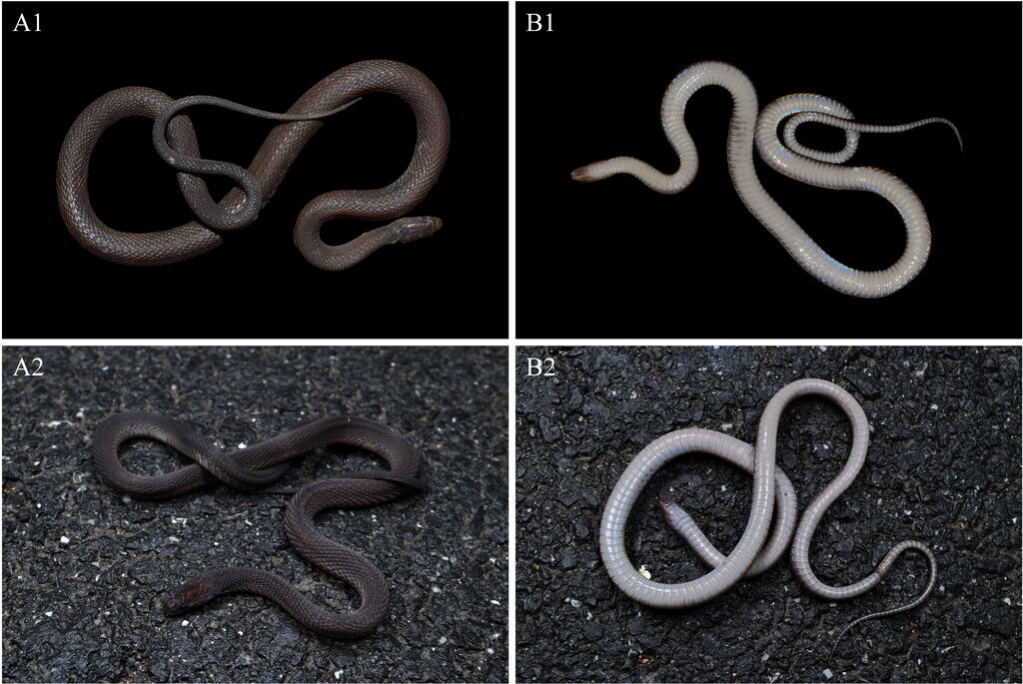
*Achalinushunanensis* in life, dorsal view (**A**) and ventral view (**B**). **A1**–**B1**: CIB 20160503, adult male; **A2–B2**: specimen QHU 2024030, adult female. **A1–B1** photos by Ke-Ji Guo, **A2– B2** photos by Yu-Hao Xu.

**Colouration in life.** In life, dorsum (head, body and tail) predominantly brownish-black, slightly tinged with iridescence. Head scales in dorsal view coloured like body. Eyes completely black, pupil vertically subelliptic. Supralabials mostly brownish-black. Mental, infralabials, chin shields and the 1^st^ ventral brownish-black. Ventral ground colour light creamy-yellow, darker on the sides, the outer one-sixth of the ventrals light brown. Ventral part of tail pale brown, gradually darkening towards the tip. The posterior margins of ventral scales are pale creamy-white (Fig. 2).

**Figure 3.**
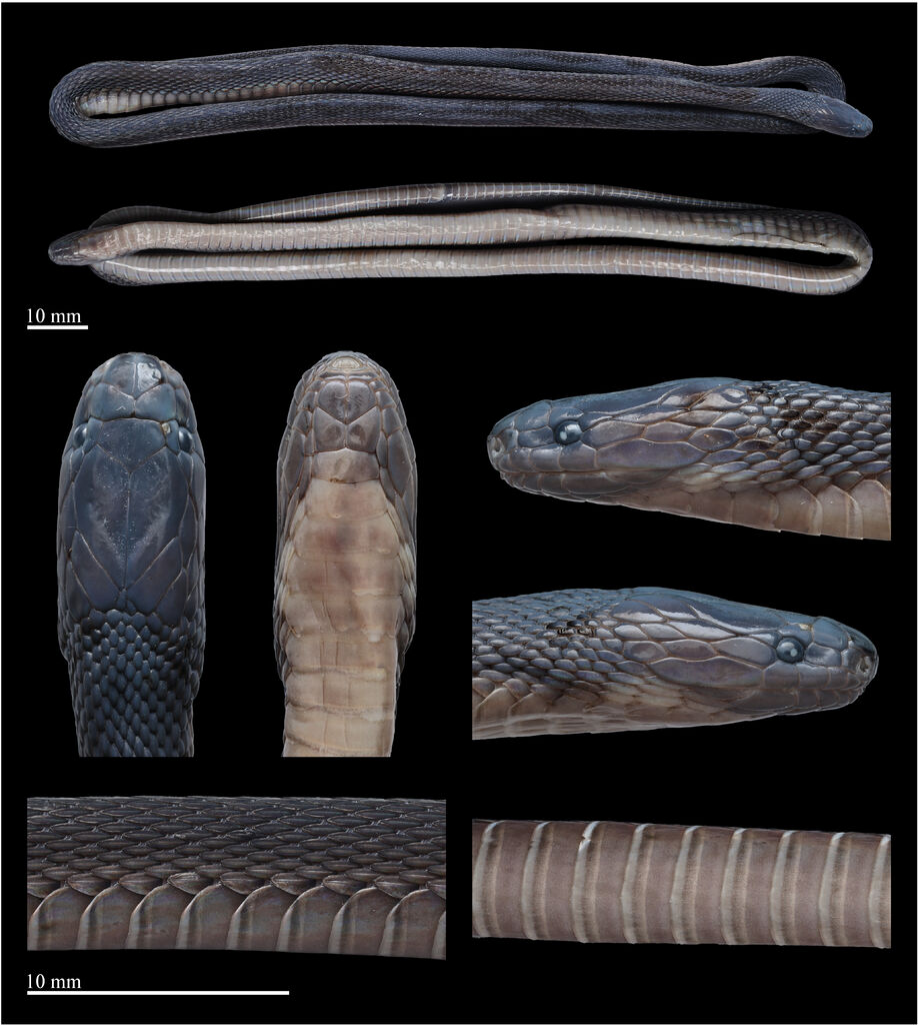
*Achalinushunanensis* (QHU 2024030), female, preserved specimen. Photos by Yu-Hao Xu. Scale bars: 10 mm.

**Colouration in preservation.** After one month preservation, the colouration still resembles the specimen in life, except that the colouration of dorsum further deepens and the background colour of the venter becomes uniform pale brownish (Fig. 3).

**Variation.** The female specimen exhibits similar colouration to the male specimens, but differs in measurements and scalation characters as follows: the examined female has a relatively larger body size (TL 428 mm vs. 262–379 mm in males); a significantly shorter tail, TAL/TL ratio 0.17 (vs. 0.22–0.24 in males); and fewer subcaudals (53 vs. 69–72 in males). The main morphological characters of *A.hunanensis* are listed in Table 2.

#### Revision of diagnostic characters

(1) dorsal scales strongly keeled, 23 rows throughout the body, the outermost row strongly keeled and substantially enlarged; (2) tail relatively short, TAL/TL ratio 0.22–0.24 in males and 0.17 in females; (3) maxillary teeth 23; (4) length of suture between internasals substantially longer than that between prefrontals, LSBI/LSBP ratio 2.0–2.1; (5) one loreal, subrectangular; (6) six supralabials, the 4^th^ and 5^th^ in contact with the eye; (7) the two anterior temporals in contact with eye; (8) ventrals 163–168 in males and about 169 in females; (9) subcaudals 69–72 in males and about 53 in females, not paired.

#### Distribution and habits

Currently, *Achalinushunanensis*is is known from Hunan Province: Anhua County, Hecheng District, Ningxiang County; and Guizhou Province: Dushan County (Fig. 1). The species was found in leaf litter in well-preserved montane evergreen broadleaf forests (550–1300 m a.s.l.).

### Achalinus yunkaiensis

Wang, Li & Wang, 2019

FDF163A9-3B41-555A-B225-9FB1759BC869

#### Materials

- **Type status:** Other material. **Occurrence:** catalogNumber: QHU 2024027; associatedSequences: GenBank: PQ281492; occurrenceID: 9FDEAC40-C043-5D14-9EE7-ECF9CD3339AE; **Taxon:** scientificName: *Achalinusyunkaiensis*; order: Squamata; family: Xenodermidae; genus: Achalinus; **Location:** country: China; stateProvince: Guizhou; county: Xifeng; verbatimElevation: 1300 m; verbatimCoordinates: 27°09'07"N, 106°37'49"E; **Event:** eventDate: 07-06-2024
- **Type status:** Other material. **Occurrence:** catalogNumber: ZTY-r 2023003; occurrenceID: 33281C61-8089-5F65-BFF2-7C5FB93F6835; **Taxon:** scientificName: *Achalinusyunkaiensis*; order: Squamata; family: Xenodermidae; genus: Achalinus; **Location:** country: China; stateProvince: Hunan; county: Dongan; verbatimElevation: 871 m; verbatimCoordinates: 26°40'56"N, 111°18'50"E; **Event:** eventDate: 25-07-2023

#### Description of the specimen from Guizhou Province (QHU 2024027)

**Measurements and scalation.** The measurements and scalation characters of *A.yunkaiensis* are listed in Table 3. Body slender and cylindrical; head slightly wider than neck; eye small, ED 0.9 mm; rostrum small, triangular, slightly visible from above; the suture between internasals subequal to the suture between prefrontals, LSBI 1.3 mm, LSBP 1.2 mm, LSBI/LSBP ratio 1.1; nasal divided into two sections by nasal cleft, nostril in the anterior part of the nasal; prefrontals paired; frontal one, pentagonal, pointed to the rear, slightly wider than high, much shorter than the parietals; one loreal, subrectangular, wider than high, exact measurements are impossible due to injury; supraocular one, pentagonal; TEMP 8/7, arranged in three rows (2+2+4 in left and 2+2+3 in right), the anterior two in contact with the eye; six supralabials, the 4^th^–5^th^ contacting the eye, the last one much elongated; two pairs of chin shields, the anterior pair almost equal to the posterior pair, followed by preventrals; one mental; 6/5 infralabials, the first pair in contact with each other after the mental and before the 1^st^ chin shields, 1^st^–4^th^ touching the first pair of chin shields on left and 1^st^–3^rd^ touching the first pair of chin shields on the right.

Dorsal scales strongly keeled, 23 rows throughout the body, the outermost row smooth and substantially enlarged. VS 146; anal entire; SC 59, not paired.

**Figure 4.**
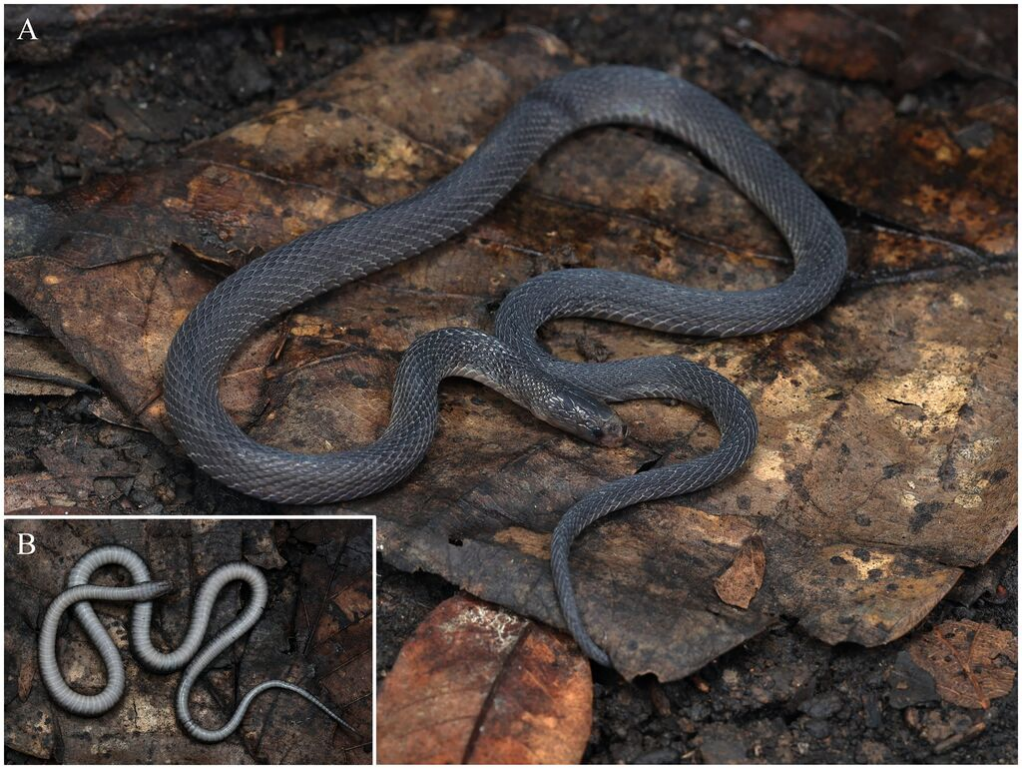
Dorsal view (**A**) and ventral view (**B**) of *Achalinusyunkaiensis* (QHU 2024027) from Xifeng County, Guizhou Province in life. Photos by Yu-Hao Xu.

**Colouration in life.** In life, all scales tinged weakly iridescent. Dorsum brown and the five innermost dorsal scale rows a little darker, forming an inconspicuous longitudinal vertebral line from posterior margin of the parietals to tail tip. Head scales in dorsal view the same colour as the dorsum and dorsal darker than lateral. Eyes completely black, pupil vertically subelliptic. Supralabials mostly brownish. Mental, infralabials, chin shields brown. Ventral ground colour of body and tail generally pale greyish-white and darker laterally. The posterior margins of ventral scales are greyish-white (Fig. 4).

**Figure 5.**
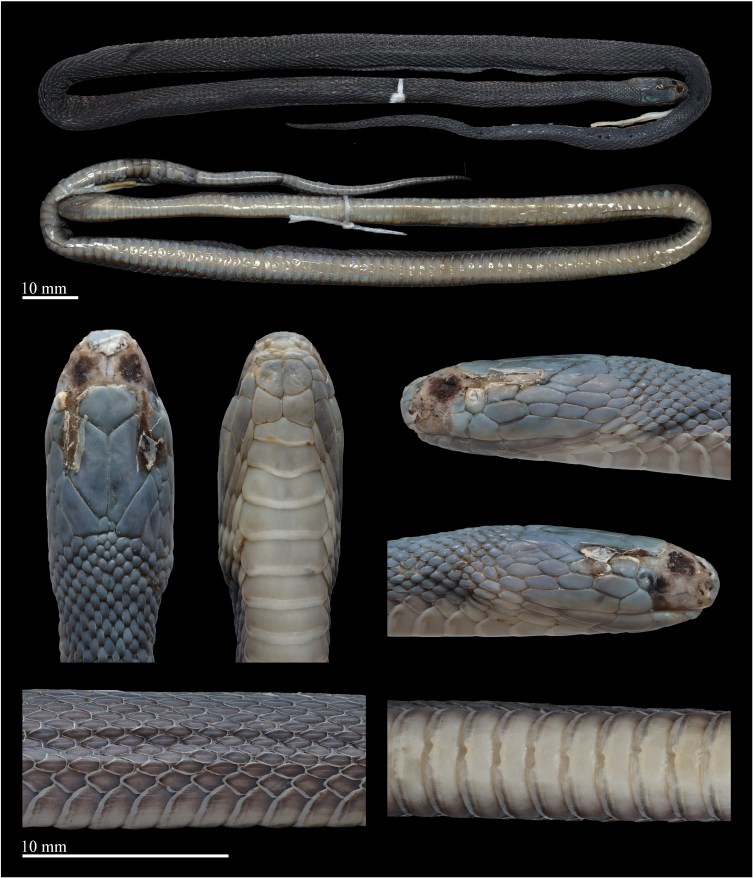
Preserved specimen of *A.yunkaiensis* (QHU 2024027) from Xifeng County, Guizhou Province. Photos by Yu-Hao Xu. Scale bars: 10 mm.

**Colouration in preservation.** In preservation, colouration darkens. Dorsum taupe, the longitudinal vertebral line almost disappearing. The colouration of venter is fading and becomes pale brownish-grey (Fig. 5).

#### Distribution and habits

*Achalinusyunkaiensis* is currently known from several locations in China, including Maoming City, Guangdong Province; Guilin City, Guangxi Zhuang Autonomous Region; Yongzhou and Shaoyang City, Hunan Province; Guiyang City, Guizhou Province; and Luzhou City, Sichuan Province, at elevations ranging from 425 to 1600 m a.s.l. (Fig. 1). The species usually prefers to hide under rocks, decaying wood or fallen leaves and the surrounding environment is untouched subtropical monsoon forests.

## Analysis

**Figure 6.**
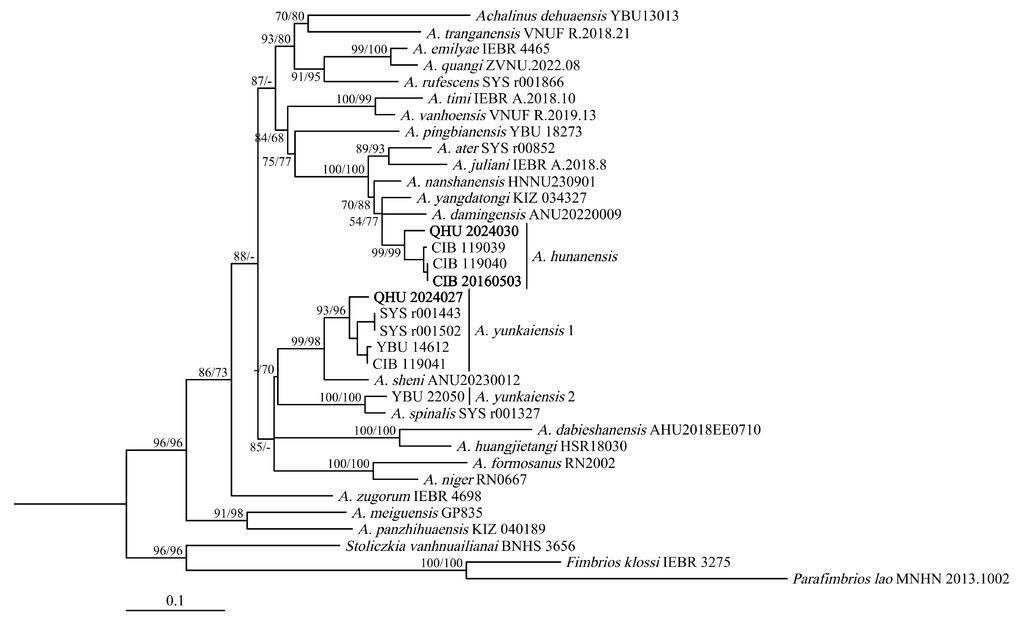
Maximum-likelihood tree of the genus *Achalinus* inferred from the *CO1* gene fragment. The nodes supporting values on branches are presented as SH-like approximate likelihood ratio test (SH)/Ultrafast Bootstrap Approximation (UFB), the ones lower than 50 being displayed as “–”.

The topology obtained by Maximum Likelihood analysis is shown in Fig. 6, based on the *CO1* genes with a total length of 621 base pairs (bp). All *Achalinus* specimens clustered into one monophyletic group with strong support (SH 96/UFB 96). The specimens QHU 2024030 and CIB 20160503 formed a clade with the *A.hunanensis* type series (SH 99/UFB 99), with a low intraspecific generic divergence ranged from 0.0%–3.4% (Table 4), which is less than the minimum interspecific uncorrected *p*-distance amongst other recognised species of *Achalinus*. Molecular and morphological analyses indicate that the two specimens should be identified as *A.hunanensis*. In addition, the specimen QHU 2024027 formed a clade with *A.yunkaiensis* in concordance with the morphological results.

## Discussion

Molecular phylogenetic analysis revealed that sequence OR344062 (voucher number: YBU 22050) clustered with *A.spinalis* with high support (SH 100/UFB 100) and the uncorrected *p*-distance was only 2.8%. However, Zhang et al. (2023) reported that this sequence formed a clade with the *A.yunkaiensis* type series, with low intraspecific genetic divergence ranging from 3.1% to 3.3%. Meanwhile, according to the data provided by Zhang et al. (2023), the female specimen YBU 22050 from Luzhou City, Sichuan Province shows the following distinctive morphological characters compared to the known female specimens of *A.yunkaiensis*: (1) tail relatively long, TaL/TL ratio 0.24 (vs. 0.16–0.20); (2) VS 145, SC 65, VS+SC 210 (vs. VS 144–156, SC 51–55, VS+SC 195–205); (3) length of suture between internasals significantly shorter than that between prefrontals, LSBP 17.7 mm, LSBI 11.6 mm, LSBI/LSBP ratio 0.66 (vs. LSBI = LSBP). This discrepancy is notably perplexing. Therefore, comprehensive sampling of both morphological and genomic data is necessary to better understand the population or species structure within *A.yunkaiensis*.

Recent studies using a combination of morphological and molecular analyses have revealed significant genetic divergences within the genus *Achalinus*, suggesting that the diversity of this genus has been greatly underestimated and many species seem to have very limited distributions (Wang et al. 2019, Ziegler et al. 2019, Miller et al. 2020, Hou et al. 2021, Ha et al. 2022, Yang et al. 2023, Zhang et al. 2023, Ma et al. 2023b, Ma et al. 2023c, Xu et al. 2024a, Xu et al. 2024b). However, the elusive lifestyles of *Achalinus* species have made it difficult for researchers to accurately assess their populations and distributions, posing challenges for conservation efforts. Currently, while *A.hainanus* Huang, 1975 is listed as "Vulnerable" (VU) and *A.werneri* Van Denburgh, 1912 as "Near Threatened" (NT) by the IUCN Red List of Threatened Species, the conservation status of most *Achalinus* species remains largely unassessed (Kidera and Ota 2017, Lau and Zhou 2017). Given their restricted ranges and vulnerability to overexploitation, all these species might need to be reclassified into higher threat categories following careful evaluation.

Previously, *A.hunanensis* was known exclusively from its type locality Hunan Province, with the holotype collected at an altitude of 880 m and the paratype at 1200 m (Ma et al. 2023b). However, the discovery of the female specimen QHU 2024030 at an altitude of 1300 m in Dushan County, Guizhou Province and the male specimen CIB 20160503 at 550 m in Anhua County, Hunan Province, substantially extends the known geographical and elevational range of the species. Additionally, the identification of *A.yunkaiensis* in Guiyang City also represents the first record of this species in Guizhou Province. The findings of this study increase the number of recorded *Achalinus* species in Guizhou Province to five and illustrate that the diversity of the genus in Guizhou may still be highly underestimated and additional surveys are required to understand the true diversity of *Achalinus* in southwest China.

## Supplementary Material

XML Treatment for Achalinus hunanensis

XML Treatment for Achalinus yunkaiensis

## Figures and Tables

**Table 1.** Localities, voucher information, GenBank numbers and references for samples used in this study.

**NO.**	**Species name**	**Locality**	**Voucher NO.**	**GenBank No.**	**References**
1	*Achalinushunanensis*	Dushan, Guizhou, China	QHU 2024030	PQ281493	Xu et al. (2024b)
2	*A.hunanensis*	Anhua, Hunan, China	CIB 20160503	PQ281494	This study
3	*A.hunanensis*	Hecheng, Hunan, China	CIB 119039	OQ848425	Ma et al. (2023b)
4	*A.hunanensis*	Ningxiang, Hunan, China	CIB 119040	OQ848426	Ma et al. (2023b)
5	*A.yunkaiensis*	Xifeng, Guizhou, China	QHU 2024027	PQ281492	Xu et al. (2024b)
6	*A.yunkaiensis*	Luzhou, Sichuan, China	YBU 22050	OR344062	Zhang et al. (2023)
7	*A.yunkaiensis*	Dawuling Forestry Station, Guangdong, China	SYS r001443	MN380329	Wang et al. (2019)
8	*A.yunkaiensis*	Dawuling Forestry Station, Guangdong, China	SYS r001502	MN380330	Wang et al. (2019)
9	*A.yunkaiensis*	Maoershan Nature Reserve, Guangxi, China	YBU 14612	MT365525	Yu et al. (2020)
10	*A.yunkaiensis*	Xinning, Hunan, China	CIB 119041	OQ978852	Ma et al. (2023a)
11	*A.ater*	Huaping Nature Reserve, Guangxi, China	SYS r00852	MN380334	Wang et al. (2019)
12	*A.dabieshanensis*	Yaoluoping Nature Reserve, Anhui, China	AHU2018EE0710	MW316598	Zhang et al. (2023)
13	*A.damingensis*	Nanning, Guangxi, China	ANU20220009	OP644487	Yang et al. (2023)
14	*A.dehuaensis*	Dehua, Fujian, China	YBU 13013	MZ442662	Li et al. (2021)
15	*A.emilyae*	Hoanh Bo, Vietnam	IEBR 4465	MK330857	Ziegler et al. 2019
16	*A.formosanus*	Taiwan, China	RN2002	KU529452	Unpublished
17	*A.huangjietangi*	Huangshan, Anhui, China	HSR18030	MT380191	Huang et al. (2021)
18	*A.juliani*	Ha Lang, Cao Bang, Vietnam	IEBR A.2018.8	MK330854	Ziegler et al. (2019)
19	*A.meiguensis*	Mianyang, Sichuan, China	GP835	MZ442641	Li et al. (2021)
20	*A.nanshanensis*	Huaihua, Hunan Province, China	HNNU230901	OR523368	Li et al. (2024)
21	*A.niger*	Taiwan, China	RN0667	KU529433	Unpublished
22	*A.panzhihuaensis*	Yanbian, Sichuan, China	KIZ 040189	MW664862	Hou et al. (2021)
23	*A.pingbianensis*	Honghe, Yunnan, China	YBU 18273	MT365521	Li et al. (2020)
24	*A.quangi*	Phu Yen, Son La, Vietnam	ZVNU.2022.08	OQ197471	Pham et al. (2023)
25	*A.rufescens*	Hongkong, China	SYS r001866	MN380339	Wang et al. (2019)
26	*A.sheni*	Lianyuan, Hunan, China	ANU20230012	OR178145	Ma et al. (2023c)
27	*A.spinalis*	Badagong Mountains, Hunan, China	SYS r001327	MN380340	Wang et al. (2019)
28	*A.timi*	Thuan Chau, Son La, Vietnam	IEBR A.2018.10	MK330856	Ziegler et al. (2019)
29	*A.tranganensis*	Ninh Binh, Vietnam	VNUF R.2018.21	MW023086	Luu et al. (2020)
30	*A.vanhoensis*	Van Ho, Son La, Vietnam	VNUF R.2019.13	ON677935	Ha et al. (2022)
31	*A.yangdatongi*	Wenshan Nature Reserve, Yunnan, China	KIZ 034327	MW664865	Hou et al. (2021)
32	*A.zugorum*	Bac Me, Ha Giang, Vietnam	IEBR 4698	MT502775	Miller et al. (2020)
	Out group				
33	*Fimbriosklossi*	Quang Ngai, Vietnam	IEBR 3275	KP410744	Teynié et al. (2015)
34	*Parafimbrioslao*	Louangphabang, Laos	MNHN 2013.1002	KP410746	Teynié et al. (2015)
35	*Stoliczkiavanhnuailianai*	Mizoram, India	BNHS 3656	OL422476	Deepak et al. (2021)

**Table 2.** Morphological variation of *Achalinushunanensis* obtained from specimens examined in this study and Ma et al. (2023b).

**Voucher number**	**QHU 2024030**	**CIB 20160503**	**CIB 119039**	**CIB119040**
**Location**	Dushan, Guizhou	Anhua, Hunan	Hecheng, Hunan	Ningxiang, Hunan
**Sex**	♀	♂	♂	♂
**SVL**	355	288	255	204
**TL**	428	379	329	262
**TAL**	73	91	74	58
**TAL/TL**	0.17	0.24	0.23	0.22
**HL**	12.5	12.7	7.9	6.3
**HW**	5.9	4.6	4.8	3.4
**SL**	3+2+1	3+2+1	3+2+1	3+2+1
**IL**	6/6	5/5	5/6	5/5
**Chin**	2	2	2	2
**IL–1stChin**	3/3	3/3	3/4	3/3
**Lor**	1	1	1	1
**LorH**	1.0	1.2	1.0	0.9
**LorL**	1.6	1.7	1.5	1.5
**LorH/LorL**	0.63	0.71	0.67	0.60
**LSBI**	1.9	1.8	1.78	1.52
**LSBP**	0.9	0.9	0.88	0.76
**LSBI/LSBP**	2.11	2.00	2.02	2.00
**LSBI vs. LSBP**	>	>	>	>
**ED**	1.2	1.1	1.4	1.4
**TEMP**	2+2+3/2+2+3	2+3+4/2+3+4	2+2+4/2+2+4	2+2+4/2+2+4
**aTEMP-Eye**	2/2	2/2	2/2	2/2
**SPO**	1	1	1	1
**DSR**	23-23-23	23-23-23	23-23-23	23-23-23
**VS**	169	168	163	165
**CP**	1	1	1	1
**SC**	53	69	69	72

**Table 3.** Morphological variation of *Achalinusyunkaiensis* obtained from specimens examined in this study, Wang et al. (2019), Yu et al. (2020), Ma et al. (2023a), and Zhang et al. (2023).

	**Location**						
	Guizhou	Guangxi	Sichuan	Hunan		Guangdong	
**Sex**	♂	♀	♀	♂	♀	♂	♀
**N**	1	1	1	1	1	4	1
**SVL**	235	286	232	247	204	189–359	386
**TL**	306	339	306	322	256	232–418	448+
**TAL**	71	53	74	75	52	43–63	62+
**TAL/TL**	0.23	0.16	0.24	0.23	0.20	0.19–0.20	–
**SL**	3+2+1	3+2+1	3+2+1	3+2+1	3+2+1	3+2+1	3+2+1
**IL**	5 or 6	6	6	6	6	6	6
**Chin**	2	2	2	2	2	2	2
**IFL–1stChin**	1^st^–3^rd^ or 4^th^	1^st^–3^rd^	1^st^–3^rd^	1^st^–4^th^	1^st^–3^rd^	1^st^–3^rd^	1^st^–3^rd^ or 4^th^
**Lor**	1	1	1	1	1	1	1
**LorH**	–	–	0.6	0.8	0.74	0.8–1.3	1.2
**LorL**	–	–	1.2	1.7	1.51	1.3–2.2	2.2
**LorH/LorL**	–	–	0.5	0.47	0.49	0.56–0.64	0.55
**LSBI vs. LSBP**	=	=	<	=	=	=	=
**TEMP**	2+2+3 or 2+2+4	2+2+3 or 2+2+4	2+2+3	2+2+3	2+2+3	2+2+3 or 2+2+4	2+2+3
**aTEMP-Eye**	2/2	2/2	2/2	2/2	2/2	2/2	2/2
**SPO**	1	1	1	1	1	1	1
**DSR**	23-23-23	23-23-23	23-23-23	23-23-23	23-23-23	23-23-23	23-23-23
**VS**	146	151	145	153	150	144–156	156
**CP**	1	1	1	1	1	1	1
**SC**	59	51	65	61	55	51–55	38+
**VS+SC**	205	202	210	214	205	195–205	–

**Table 4.** Uncorrected *p*-distances (%) amongst the *Achalinus* species, based on partial mitochondrial *CO1* gene for species compared in this study.

**ID**	**Species**	**1–4**	**5–9**	**10**	**11**	**12**	**13**	**14**	**15**	**16**	**17**	**18**	**19**	**20**	**21**	**22**	**23**	**24**	**25**	**26**	**27**	**28**	**29**	**30**	**31**
1–4	*A.hunanensis*	0–3.4																							
5–9	*A.yunkaiensis* 1	11.6–12.3	0–3.2																						
10	*A.yunkaiensis* 2	14.0	11.2–11.9	–																					
11	*A.ater*	7.0–7.6	11.7–12.9	14.0	–																				
12	*A.dabieshanensis*	16.7–17.2	15.0–15.9	16.1	14.8	–																			
13	*A.damingensis*	5.7–6.4	12.1–12.9	15.0	7.4	15.9	–																		
14	*A.dehuaensis*	15.3–15.7	13.3–14.8	14.4	16.1	18.6	15.2	–																	
15	*A.emyliae*	12.9–13.8	12.7–13.1	14.4	11.2	18.0	12.9	15.3	–																
16	*A.formosanus*	13.6–13.8	11.7–12.5	13.3	13.3	18.8	14.2	15.7	13.6	–															
17	*A.huangjietangi*	15.0–15.3	13.3–14.2	14.2	13.1	11.0	15.2	15.3	15.5	16.1	–														
18	*A.juliani*	8.7–9.1	11.4–12.9	13.4	6.6	15.9	8.3	14.8	12.9	11.4	14.4	–													
19	*A.meiguensis*	16.1–16.3	15.3–15.9	16.7	15.3	18.0	16.5	18.4	15.3	15.5	16.9	16.7	–												
20	*A.niger*	13.3	10.4–11.9	13.3	12.9	16.1	13.3	16.3	12.7	8.5	15.7	11.7	13.8	–											
21	*A.nanshanensis*	4.9–5.7	11.0–12.5	14.0	6.8	16.1	5.1	13.4	13.3	13.6	14.6	8.1	17.6	12.1	–										
22	*A.panzhihuaensis*	16.5–16.9	15.7–16.1	15.9	16.5	16.5	15.5	15.5	16.5	16.1	15.7	15.7	11.4	14.0	15.3	–									
23	*A.pingbianensis*	10.6–11.0	10.8–11.6	13.3	11.0	15.3	10.2	14.6	13.1	14.2	14.0	11.6	16.7	11.9	11.0	14.8	–								
24	*A.quangi*	12.1–13.6	12.7–13.6	13.3	11.4	18.4	12.7	15.5	2.8	13.6	15.9	12.5	15.2	12.1	12.7	16.9	13.6	–							
25	*A.rufescens*	11.7	11.6–13.4	12.9	11.7	15.9	0.0	12.9	9.7	13.8	14.6	11.2	18.6	13.8	11.4	15.9	12.7	10.0	–						
26	*A.sheni*	12.7–12.9	6.4–6.8	11.6	13.1	15.9	13.3	13.8	13.6	12.7	12.9	13.6	14.2	12.9	14.0	14.8	11.2	14.2	13.1	–					
27	*A.spinalis*	14.0–14.4	10.8–11.9	2.8	14.6	16.5	14.6	14.2	14.4	14.2	14.4	14.0	15.9	13.8	14.4	16.1	13.3	13.6	12.7	11.7	–				
28	*A.timi*	12.1–12.3	12.7–13.3	13.8	12.7	16.5	12.5	15.0	12.9	13.3	15.9	13.4	15.9	11.6	13.1	15.3	11.9	12.5	14.0	13.3	14.0	–			
29	*A.tranganensis*	14.0–14.8	12.3–14.0	15.2	12.5	15.3	13.8	14.0	12.3	16.9	13.4	14.2	16.3	14.6	13.4	16.5	13.4	11.7	12.7	14.2	15.5	13.4	–		
30	*A.vanhoensis*	11.2–11.4	11.6–12.3	11.9	11.9	15.5	11.7	14.8	11.7	13.6	15.2	12.7	15.7	11.7	11.9	15.3	10.6	11.6	12.9	12.7	12.3	4.5	11.9	–	
31	*A.yangdatongi*	5.1–5.5	11.7–12.5	13.3	6.4	16.7	5.7	14.4	12.7	14.2	14.8	7.6	17.2	13.4	4.5	15.7	10.8	12.5	11.6	14.0	14.2	12.7	12.9	10.8	–
32	*A.zugorum*	11.9–12.1	10.4–11.9	12.9	13.3	15.3	12.3	14.2	12.9	13.4	15.0	13.3	15.0	13.1	12.7	15.2	10.2	13.1	13.8	10.4	13.4	13.4	11.7	11.7	12.1
